# CSN6 aggravates inflammation and Myocardial injury in macrophage of sepsis model by MIF

**DOI:** 10.1038/s41598-025-07339-1

**Published:** 2025-07-01

**Authors:** Qianying Song, Changming Zhou, Yufei Liu, Liping Wang, Huiyi Lv, Cuiying Zhang, Xuanqi Wang

**Affiliations:** Second Affiliated Hospital of Dalian Medical University, Dalian, China

**Keywords:** CSN6, Myocardial injury, Macrophage, Sepsis, MIF, Molecular biology, Cardiology

## Abstract

Sepsis, one of the leading causes of death in critically ill patients, is characterized by multiple organ dysfunction due to a dysregulated immune response to infection. Caregivers closely monitor patients’ organ function indicators in the intensive care unit,which is essential for the early identification and management of organ dysfunction cauxsd by sepsis. Hence, we investigated the effects of CSN6 on sepsis and its underlying mechanism. RAW264.7 cell inducted with lipopolysaccharide (LPS) and adenosine triphosphate (ATP). CSN6 protein expression increased in an in vitro model of sepsis. We collected samples from 10 sepsis patients (It was collected under strict compliance with ethical norms and nursing procedures) and performed single-cell analysis for CSN6 expression. CSN6 aggravated macrophage inflammation in an in vitro model of sepsis. CSN6 aggravated macrophage ferroptosis in an in vitro model of sepsis. CSN6 aggravates mitochondrial damage in an in vitro model of sepsis. CSN6 induces MIF expression in macrophages in an in vitro model of sepsis. MIF inhibitors reduced the effects of CSN6 on inflammation and ferroptosis in an in vitro sepsis model. CSN6 protein at 11-ARG, 21-ARG, 31-LEU, and 32-ASP linked to MIF protein at 280-ASN and 366-SER. In conclusion, CSN6 appears to aggravate inflammation in macrophages in a sepsis model via MIF signaling. This finding suggests that future therapeutic strategier targeting the CSN6 and MIF pathways may require nurses to closely monitor changes in inflammatory responses and potential treatment side effecta at the bedside. Further research involving in vivo models, such as examining CSN6 and MIF expression levels in macrophages or monocytes from sepsis and control mice, is essential to fully confirm these findings and establish the therapeutic potential of targeting the CSN6/MIF axis in sepsis. The nursing research team plays a key role in translating basic research findings into clinical practice,including the developpment of early warning tools and individualized management programs based on these biomarkers.

## Introduction

Sepsis is a life-threatening organ dysfunction caused by an imbalance in the host’s systemic inflammation and immune response to infection^1^. In recent years, with the in-depth study of the pathogenesis of sepsis, research on intestinal dysfunction in sepsis has gradually become a hot topic and is considered the “engine” of multiple organ failure^2^. Although studies have shown that immediate initiation of anti-infection and supportive treatment measures can significantly improve the prognosis of critically ill patients, the incidence and mortality rates remain high, with 48.9 million cases of sepsis and 11 million sepsis-related deaths worldwide each year^3^. Therefore, identifying new rapid and effective biomarkers may help diagnose sepsis and assess the prognosis of patients with sepsis. In addition, it may contribute to the understanding of the pathogenesis of sepsis^4^.

Sepsis, one of the leading causes of death in critically ill patients, is characterized by multiple organ dysfunction due to a dysregulated immune response to infection^5^. Strengthening the prevention, diagnosis, and management of sepsis is an urgent clinical issue. Sepsis is often accompanied by various organ injuries such as cardiac and renal injuries^6^. Among them, cardiac injury is an important factor that worsens the condition caused by organ damage, as endotoxins damage the myocardium through various pathways, causing myocardial injury, and various cytokines also participate in the entire pathological process, such as tumor necrosis factor-alpha and interleukins, further exacerbating myocardial cell membrane permeability^7^. Although drugs such as dexamethasone can effectively inhibit the inflammatory response and serve as major inhibitors and therapeutic drugs, their side effects are significant and they do not significantly improve the survival rate of critically ill patients, thereby reducing the safety of sepsis-induced myocardial injury treatment^8^.

The macrophage migration inhibitory factor (MIF) is a pleiotropic cytokine that promotes inflammation and carcinogenesis^9^. It is released by various types of immune cells, including monocytes, macrophages, neutrophils, T cells, B cells, dendritic cells, and eosinophils^9^. It can also be secreted by certain endocrine, endothelial, and epithelial cells in response to inflammatory stimuli or injury^9^. MIF was first discovered in 1966 as a mediator of delayed hypersensitivity reactions and is considered a key participant in innate immune responses^10^. It triggers inflammatory responses by stimulating the production of pro-inflammatory factors such as tumor necrosis factor-alpha (TNF-α), interferon gamma (IFN-γ), interleukin 1β (IL-1β), IL-6, IL-17, and prostaglandin E2^11^. As research has progressed, MIF has been shown to promote tumor growth and tumor immunity processes, including differential expression of inflammatory reactions, cytokines, and chemokines, as well as activation of immune checkpoint ligands and receptors^12^.

Constitutive photomorphogenesis 9 signalosome subunit 6 (CSN6) is a highly conserved multiprotein complex that regulates protein degradation and plays an important role in regulating cell proliferation, signal transduction, transcriptional activation, apoptosis, and DNA damage repair in all eukaryotes during evolution^13–15^. Hence, we investigated the effects of CSN6 on sepsis and its underlying mechanism.

## Materials and methods

### Sepsis model

RAW264.7 cells (murine macrophage cells) were purchased from the Shanghai Cell Bank of the Chinese Academy of Sciences (Shanghai, China) and maintained in DMEM (Gibco), supplemented with 10% FBS (Gibco), in a humidified 5% (v/v) CO2 atmosphere at 37 °C. Transfection was performed using Lipofectamine 2000 (Thermo Fisher Scientific, USA). After 48 h of transfection, RAW264.7 cells were exposed to both LPS (100 ng/mL for 4 h) and ATP (2 mM for 30 min) to mimic the inflammatory and pyroptotic conditions of sepsis. LPS stimulates a strong inflammatory response in macrophages by activating the TLR4 pathway, leading to the production of pro-inflammatory cytokines such as TNF-α and IL-6. ATP, in contrast, acts as a secondary signal that activates the NLRP3 inflammasome, driving pyroptosis and further cytokine release.To better understand the individual contributions of these stimuli, we included experiments that separately examined the effects of LPS and ATP. The results showed that LPS alone predominantly induced inflammatory cytokine production, whereas ATP alone triggered moderate inflammasome activation without significantly affecting cytokine levels. Combined stimulation with LPS and ATP more closely resembles the complex inflammatory environment seen in sepsis, where both cytokine release and inflammasome activation are present, providing a more comprehensive model of sepsis-induced macrophage dysfunction.

By using both LPS and ATP, we enhanced the in vitro sepsis model to reflect both the immune activation and cell death mechanisms that are hallmark features of sepsis, offering a more accurate representation of pathophysiological conditions.

### Human samples and ethical approval

Patient samples were collected from 10 individuals diagnosed with sepsis according to the Sepsis-3 criteria at our hospital between January 2023 and December 2023. The study protocol was approved by the Ethics Committee of our institution (approval number: ETH-2022–018). Written informed consent was obtained from all participants or their legal representatives before sample collection. Patient confidentiality was maintained throughout the study, and all procedures were performed in accordance with the Declaration of Helsinki. Demographic and clinical information was collected but de-identified to protect patient privacy.

### RT-PCR

Cells were extracted from total RNA using RNAiso Plus reagent (Takara, Japan). cDNA was synthesized using Superscript II Reverse Transcriptase (Applied Biosystems). mRNA expression levels were quantified by quantitative real-time PCR using SYBR Green PCR Master Mix (Takara, Japan). The following primers were used for RT-PCR.

#### CSN6:

Forward: 5'- ATCGTACTACTGAGAGACCAC-3'.

Reverse: 5'- ATCGTACGATTATACGTGTAA-3'.

#### MIF:

Forward: 5'- AGTGGTGTCCGAGAAGTCAG -3'.

Reverse: 5'- TCTCTAAACCGTTTATTTCTT -3'.

#### Collagen I:

Forward: 5'-GCTCCTCTTAGGGGCCACT-3'.

Reverse: 5'-CCACGTCTCACCATTGGGG-3'.

#### Collagen III:

Forward: 5'-CATCCAGGTCCTTGAATGGT-3'.

Reverse: 5'-AGTGGATGCCATAGCTGAGC-3'.

#### β-actin (internal control):

Forward: 5'-AGAGGGAAATCGTGCGTGAC-3'.

Reverse: 5'-CAATAGTGATGACCTGGCCGT-3'.

These primer sequences were designed to target the specific mRNA regions for each gene and were optimized for use with SYBR green-based qPCR.

### Immunofluorescence and Western blot analysis

The cells were fixed with 4% paraformaldehyde for 20 min at room temperature, permeabilized with 0.5% Triton X-100 in PBS for 15 min, and blocked with 5% BSA for 30 min at 37 °C. The cells were then treated with primary antibodies overnight at 4 °C overnight: anti-CSN6 and anti-MIF. The cells were then incubated with Cy3-conjugated goat anti-rabbit or goat anti-mouse IgG DyLight 555-conjugated secondary antibodies for 2 h at 37 °C. Nuclei were stained with DAPI and the cells were observed under a fluorescence microscope (Olympus IX71, Tokyo, Japan).

Total protein was extracted from lung or cell samples using Radio-Immunoprecipitation assay (RIPA) and PMSF reagent (1:100, Beyotime, Beijing, China). Protein lysates were separated by molecular weight on SDS-PAGE gels and transferred onto Polyvinylidene Fluoride (PVDF) membranes. The membrane was blocked with 5% non-fat milk for 2 h at room temperature and incubated overnight at 4 °C with the following primary antibodies: anti-CSN6 (1:1000, Abcam), anti-MIF (1:1000, Abcam), anti-GPX4 (1:1000, Abcam), and anti-β-actin (1:10,000, ab8226, Abcam). The primary antibodies were removed and the membranes were washed with TBST. The membranes were then incubated with secondary antibody for 2 h at room temperature. Bound antibodies were detected using enhanced chemiluminescence (ECL).

The immunoprecipitation (IP) experiment was designed to test whether CSN6 binds to MIF, and the results confirmed a specific interaction between the two proteins. This finding helps to elucidate the role of CSN6 in modulating MIF-related pathways in macrophage-mediated inflammation during sepsis.

### ELISA kits

Cell samples from each group were collected at 2000 g for 10 min at 4 °C. IL-1β, IL-6, and TNF-α kits were used to measure the cytokine levels.

### Immunoprecipitation (IP)

In the immunoprecipitation (IP) experiment, lysate protein (500 μg) from RAW264.7 cells was incubated with 2 μg of protein G agarose-conjugated antibody (16e266, Millipore, Billerica, MA, USA) at 4 °C overnight with rotation. Prior to this step, the cell lysates were pre-cleared with protein G agarose beads for 1 h at 4 °C to reduce non-specific binding. The resulting immunoprecipitates were washed five times with lysis buffer (containing 50 mM Tris–HCl pH 7.5, 150 mM NaCl, 1% NP-40, 0.5% sodium deoxycholate, and protease inhibitor cocktail). Subsequently, the samples were denatured in Laemmli buffer at 95 °C for 5 min, separated on a 10% SDS-PAGE gel, and transferred onto polyvinylidene difluoride (PVDF) membranes.

The membrane was blocked with 5% non-fat milk in TBST (TBS with 0.1% Tween-20) at room temperature for 2 h and incubated with primary antibodies against CSN6 (1:100, Abcam) and MIF (1:100, 3394, Abcam) overnight at 4 °C. After washing three times with TBST (10 min each), the membranes were incubated with horseradish peroxidase-conjugated secondary antibodies (sc-2004, sc-2005, 1:2000, Santa Cruz Biotechnology) for 1 h at room temperature. The bound proteins were visualized using an enhanced chemiluminescence (ECL) system (Promega, Madison, WI, USA). As negative controls, we performed parallel experiments with either non-specific IgG or omitted the primary antibody.

Using this method, it was observed that CSN6 binds to MIF at specific sites. Specifically, CSN6 protein residues 11-ARG, 21-ARG, 31-LEU, and 32-ASP interacted with the MIF protein residues 280-ASN and 366-SER (Fig. 8D). This interaction was further confirmed by confocal microscopy, which demonstrated the colocalization of CSN6 and MIF in macrophages. To ensure reproducibility, each IP experiment was performed in triplicate with consistent results.

### Ferroptosis analysis in RAW264.7 cells

Ferroptosis in RAW264.7 cells was evaluated using multiple assays targeting lipid peroxidation, iron concentration, glutathione (GSH) activity, GPX4 expression, and cell death. Cell proliferation was assessed using Cell Counting Kit-8 (CCK-8; Dojindo). Cells were seeded in 96-well plates, and after treatment, CCK-8 solution was added to each well, followed by incubation for 1 h at 37 °C. Absorbance was measured at 450 nm to assess cell viability, which was inversely correlated with the ferroptosis activity.

Lactate dehydrogenase (LDH) release, an indicator of lipid peroxidation and membrane integrity, was measured using an LDH Cytotoxicity Assay Kit (Beyotime, China). The supernatants were collected, centrifuged, and the absorbance was measured at 490 nm. An increased LDH release is indicative of cell membrane damage and ferroptosis.

Intracellular iron concentration was quantified using an Iron Assay Kit (Abcam, ab83366). Cell lysates were prepared and centrifuged, and the absorbance at 593 nm was measured according to the manufacturer’s instructions. Elevated iron levels are key hallmarks of ferroptosis. GSH activity was measured using a GSH Detection Kit (Beyotime, China). Cell lysates were prepared, and GSH levels were quantified by measuring the absorbance at 405 nm. A decrease in GSH level reflects increased oxidative stress, a characteristic of ferroptosis.

GPX4 expression, a key regulator of ferroptosis, was analyzed at both protein and mRNA levels. Western blotting was performed on the total protein lysates using an anti-GPX4 antibody (1:1000, Abcam) to assess GPX4 protein expression. For mRNA analysis, quantitative RT-PCR was performed as previously described, and the relative GPX4 mRNA levels were calculated using the 2^-ΔΔCt method. Finally, cell death was assessed by propidium iodide (PI) staining. RAW264.7 cells were incubated with 5 µg/mL PI solution for 15 min at 37 °C, and the percentage of PI-positive cells was determined using flow cytometry. PI-positive cells indicate a loss of membrane integrity, which is associated with ferroptotic cell death.

### Scanning electron microscopy (SEM) for mitochondrial injury evaluation

To evaluate mitochondrial injury, RAW264.7 cells were subjected to SEM analysis. Following treatment with CSN6 upregulation or si-CSN6, cells were harvested and fixed immediately to minimize ischemic time, which is critical for preserving mitochondrial morphology and avoiding artifacts. The cells were fixed with 2.5% glutaraldehyde in 0.1 M phosphate buffer (pH 7.4) for 2 h at 4 °C. Rapid fixation after treatment helped ensure accurate preservation of mitochondrial structure.

After primary fixation, the cells were post-fixed with 1% osmium tetroxide for 1 h, followed by dehydration in a graded ethanol series (30% to 100%). The samples were then dried using a critical point dryer and sputter-coated with gold–palladium to increase their conductivities.

Mitochondrial damage was evaluated based on several morphological criteria, including disruption of cristae, mitochondrial swelling, and loss of outer membrane integrity. Several images from different fields were captured to comprehensively assess damage. The ischemic time until fixation was kept to a minimum, ensuring that the observed mitochondrial injury was primarily due to the experimental conditions rather than preparation artifacts.

### Molecular docking

The molecular interaction between CSN6 and MIF was investigated via molecular docking using AutoDock Vina 1.2.3 (https://vina.scripps.edu/). The three-dimensional structures of CSN6 and MIF were retrieved from Protein Data Bank (PDB) and preprocessed by removing water molecules, adding polar hydrogens, and assigning Gasteiger charges. Docking parameters included a grid box centered on the predicted binding site, with dimensions adjusted to encompass the entire interaction region. A semi-flexible docking approach was applied, allowing ligand flexibility while maintaining receptor rigidity. The top-scoring conformation, selected based on the lowest binding energy (ΔG, kcal/mol), was subjected to structural refinement. Molecular visualization and interaction analysis were performed using PyMOL 2.5.4 (https://pymol.org/). Results were validated through triplicate docking runs to ensure reproducibility.

### Statistical analysis

Data are expressed as mean ± SD. Before performing statistical comparisons, all data sets were tested for normality using the Shapiro–Wilk test. For normally distributed data, multiple comparisons were performed using GraphPad Prism 8 to conduct one-way ANOVA, followed by Tukey’s post-hoc test. For data that did not follow a normal distribution, the non-parametric Kruskal–Wallis test was used, followed by Dunn’s post-hoc test for multiple comparisons.

For experiments involving multiple comparisons, the False Discovery Rate (FDR) correction using the Benjamini–Hochberg method was applied to control for Type I errors. Homogeneity of variance was verified using Levene’s test prior to ANOVA. When this assumption was violated, Welch’s ANOVA with Games-Howell post-hoc test was used instead.

Each experiment was performed with a minimum of n = 5 biological replicates unless otherwise specified in the figure legends. Statistical significance was set at P < 0.05. All statistical analyses were performed using GraphPad Prism 8 and SPSS 25.0.

## Results

### CSN6 expression in vitro model of sepsis and macrophage

**Fig. 1:**
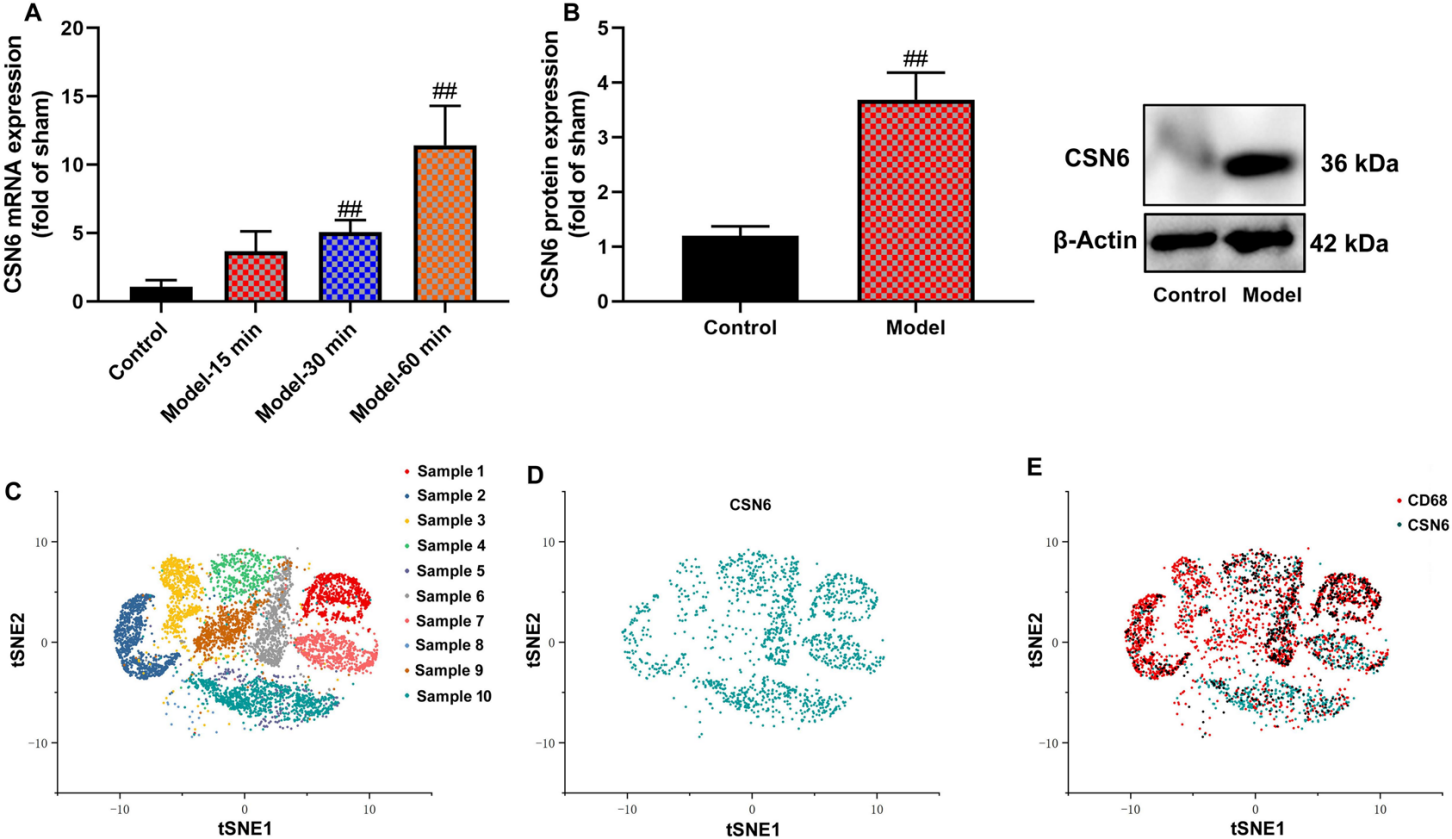
CSN6 expression in vitro model of sepsis and macrophage. CSN6 expression in an in vitro model of sepsis (**A** and **B**) and a tissue plot (**C**, **D**, **E**). ##p < 0.01 compared with to the control group.

To evaluate CSN6 expression, RAW264.7 cells were stimulated with LPS (100 ng/mL for 4 h) followed by ATP (2 mM for 30 min), which resulted in increased CSN6 mRNA expression. To provide a more detailed analysis, we examined the dose dependence of both LPS and ATP on CSN6 mRNA levels. Our findings demonstrated that CSN6 expression is dose-dependent, with increasing concentrations of LPS (ranging from 10 to 200 ng/mL) and ATP (ranging from 0.5 to 4 mM), leading to a gradual increase in CSN6 mRNA levels. Additionally, we determined that the optimal concentration for maximum CSN6 mRNA expression occurred at 100 ng/mL LPS and 2 mM ATP after 1 h of stimulation, with a peak response under these conditions. This data supports the use of these concentrations in subsequent experiments, ensuring that CSN6 expression is maximally induced for accurate evaluation of its effects in the sepsis model (Fig. 1A, n = 6 per group).

CSN6 protein expression was increased in an in vitro model of sepsis (Fig. 1B, n = 6 per group). We collected samples from 10 sepsis patients and performed single-cell analysis of CSN6 expression (Fig. 1C-E). There was a significant overlap between CD68 and CSN6, and these data showed CSN6 expression in macrophages in a model of sepsis.

### CSN6 aggravated inflammation of macrophage in vitro model of sepsis

**Fig. 2:**
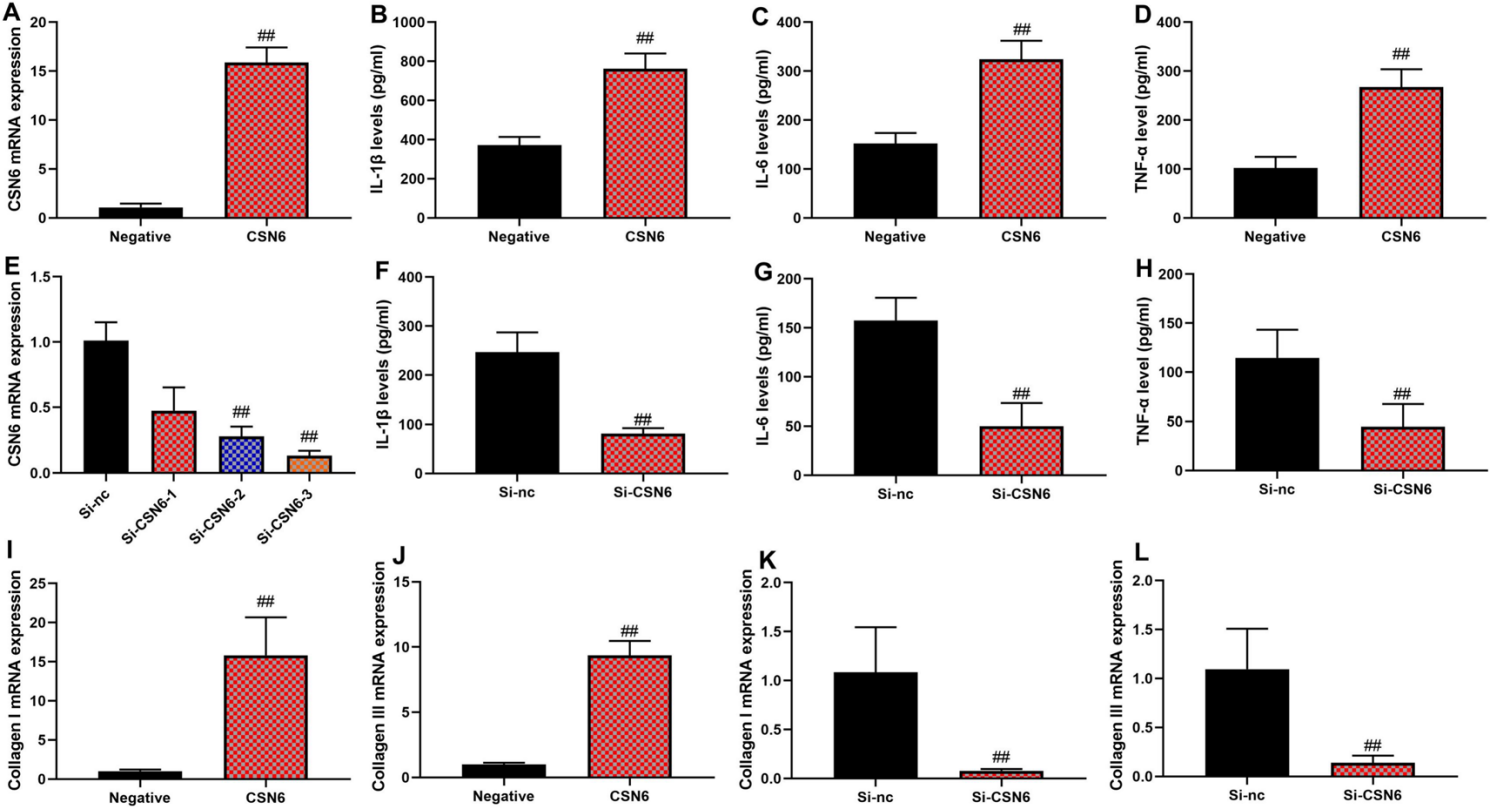
CSN6 aggravated inflammation of macrophage in vitro model of sepsis. CSN6 mRNA expression (**A**), IL-1β (**B**), IL-6 (**C**), TNF-α (**D**), and collagen I/III mRNA expression (**I** and **J**) in macrophages treated with CSN6; CSN6 mRNA expression (**E**), IL-1β (**F**), IL-6 (**G**), TNF-α (**H**) levels, and collagen I/III mRNA expression (**K** and **L**) in macrophages treated with si-CSN6. ##p < 0.01 compared with the negative or si-NC group.

In an in vitro model of sepsis, the CSN6 plasmid increased CSN6 mRNA expression, and the si-CSN6 plasmid reduced CSN6 mRNA expression (Fig. 2A, 2E, n = 6 per group). CSN6 upregulation increased IL-1β, IL-6, and TNF-α levels in an in vitro sepsis model (Fig. 2B-2D, n = n = 6 per group). Si-CSN6 reduced IL-1β, IL-6, and TNF-α levels in an in vitro sepsis model (Fig. 2F-H, n = n= 6 per group). Then, CSN6 up-regulation enhanced collagen I/III mRNA expression, and si-CSN6 effectively suppressed CSN6 expression in RAW264.7 cells in the in vitro sepsis model. To quantify this suppression, we observed that si-CSN6 reduced CSN6 mRNA levels by approximately 80% compared to control cells, achieving maximum suppression within 24 h post-transfection. Under these conditions of CSN6 suppression, collagen I/III mRNA expression was significantly reduced, reaching maximum suppression at 24 h (Fig. 2I–L,  n = 6 per group). This suggests that CSN6 plays a pivotal role in regulating collagen gene expression during the inflammatory response in sepsis models. Additionally, to enhance clarity, we have included the structure of si-CSN6 used in this study. This provides a better understanding of how si-CSN6 targets CSN6, contributing to the observed effects on collagen I/III mRNA expression.

### CSN6 aggravated ferroptosis of macrophage in vitro model of sepsis

**Fig. 3:**
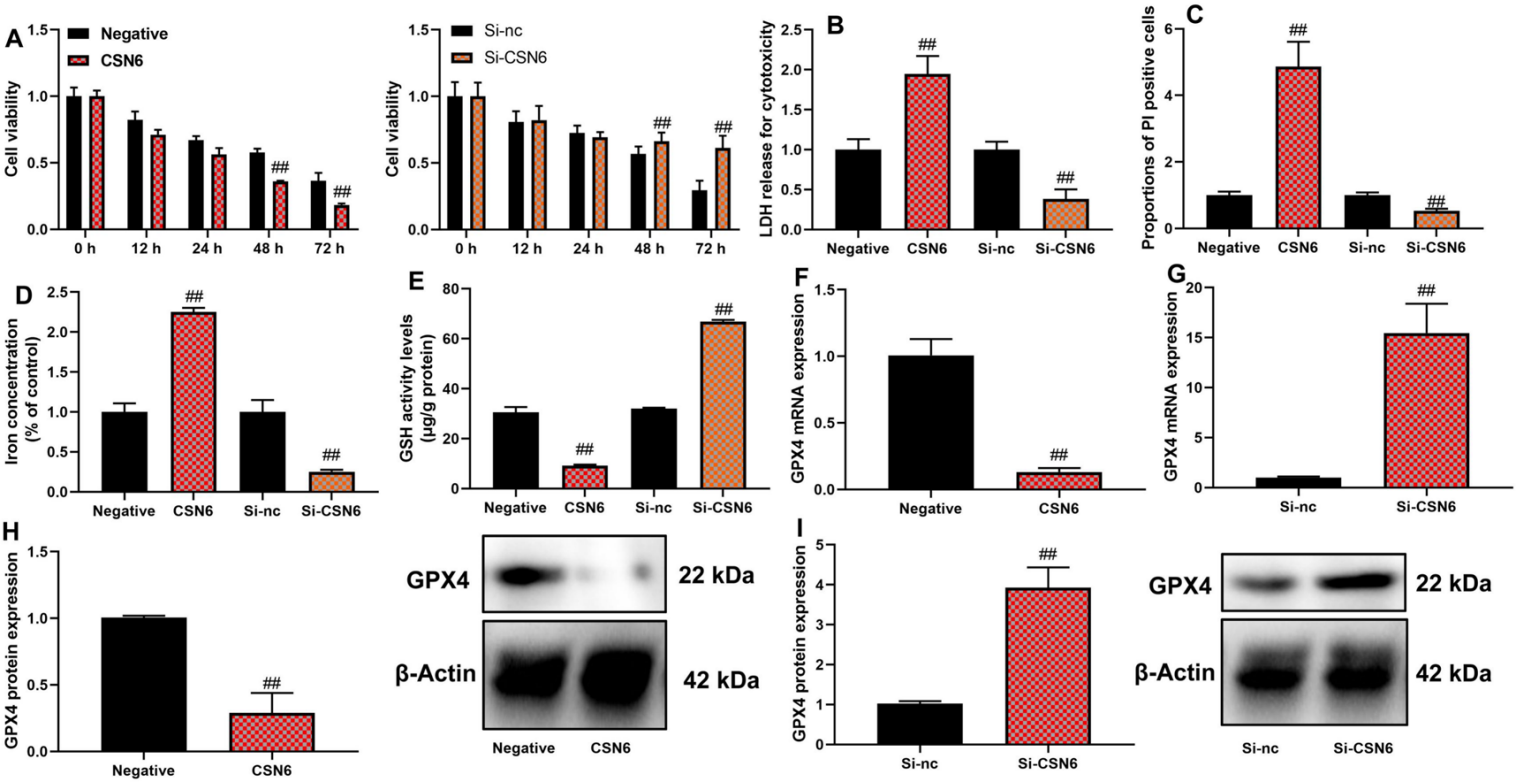
CSN6 aggravated ferroptosis of macrophage in vitro model of sepsis. (**A**) Cell proliferation, (**B**) LDH activity level, (**C**) PI-positive cells, (**C**) iron concentration, (**E**) GSH activity level, (**G**) GPX4 mRNA expression, and (**H**, **I**) GPX4 protein expression. ##p < 0.01 compared with the negative or si-NC group.

This study explored the effects of CSN6 on ferroptosis in macrophages using an in vitro sepsis model. Upregulation of CSN6 resulted in increased cell proliferation, while si-CSN6 transfection reduced cell proliferation in macrophages in the sepsis model (Fig. 3A,  n = 6 per group). Additionally, CSN6 upregulation increased lactate dehydrogenase (LDH) activity levels and the number of propidium iodide (PI)-positive cells, indicating elevated cell membrane damage, whereas si-CSN6 reduced LDH activity and the number of PI-positive cells (Fig. 3B,C, n =  5 per group).

Furthermore, CSN6 upregulation elevated intracellular iron concentration and decreased glutathione (GSH) activity, whereas si-CSN6 reduced iron concentration and restored GSH activity in macrophages (Fig. 3D, E, n =  5 per group). Moreover, CSN6 upregulation suppressed both the mRNA and protein expression of GPX4, a key regulator of ferroptosis, whereas si-CSN6 induced GPX4 mRNA and protein expression (Fig. 3F–I,  n = 6 per group).

However, a double band was observed in the negative control and CSN6 groups during the GPX4 analysis, which could have compromised the accuracy of normalization (Fig. 3, lower panel). In subsequent experiments, a single, distinct beta-actin band was presented in the Si-nc and Si-CSN6 groups, ensuring accurate normalization. This adjustment allowed for a more precise interpretation of the observed changes in CSN6 and GPX4 expression, reinforcing the conclusion that CSN6 contributes to ferroptosis in macrophages under sepsis conditions.

### CSN6 aggravated mitochondrial damage of macrophage in vitro model of sepsis

**Fig. 4:**
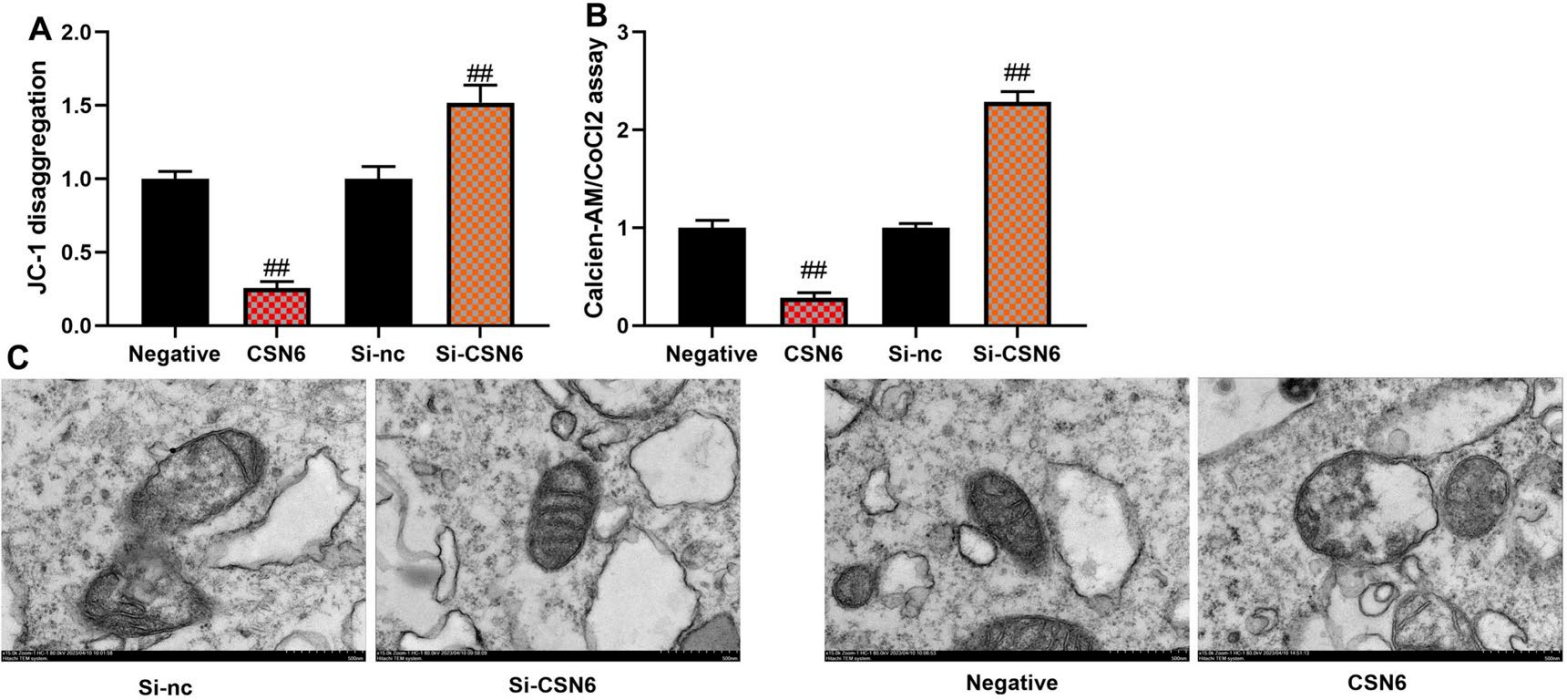
CSN6 aggravated mitochondrial damage of macrophage in vitro model of sepsis. JC-1 and calcien-AM levels (**A**, **B**) and mitochondrial damage (**C**). ##p < 0.01 compared with the negative or si-NC group.

Based on the above results, we explored the mechanism of CSN6 in ferroptosis using an in vitro model of sepsis. CSN6 upregulation reduced JC-1 and Calcien-AM levels, and si-CSN6 increased JC-1 and Calcien-AM levels in an in vitro model of sepsis (Fig. 4A, B,  n = 5 per group). Electron microscopy revealed that CSN6 upregulation reduced mitochondrial damage and si-CSN6 increased mitochondrial damage in an in vitro model of sepsis (Fig. 4C,  n = 3 per group with at least 10 cells analyzed per replicate).

### CSN6 induced MIF expression of macrophage in vitro model of sepsis

**Fig. 5:**
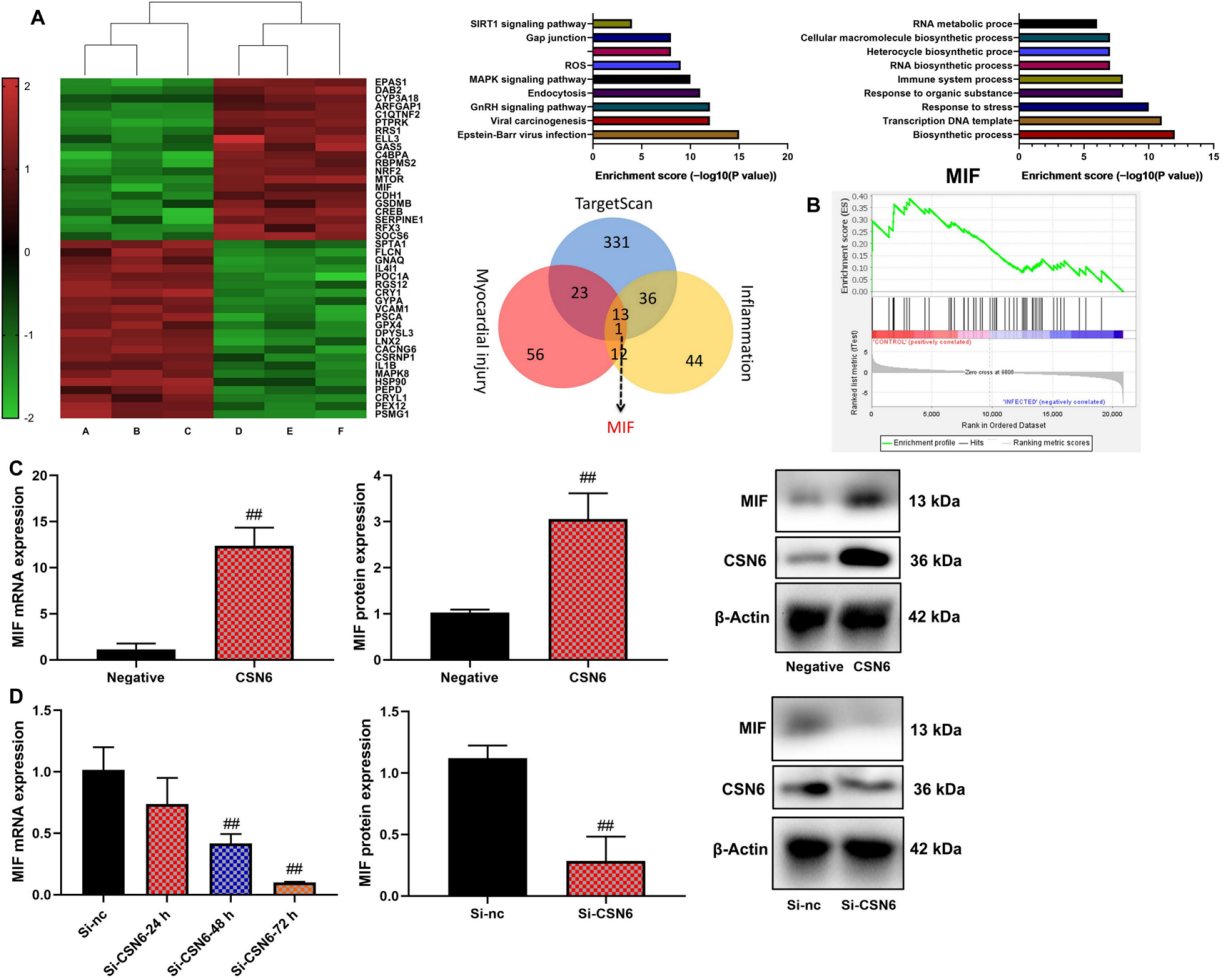
CSN6 induced MIF expression of macrophage in vitro model of sepsis. Heat map/result analysis (**A**), GSEA (**B**), CSN6/ MIF protein expression (**C** and **D**). ##p < 0.01 compared with the negative or si-NC group.

Building on previous results, we investigated the mechanism by which CSN6 contributes to mitochondrial damage in macrophages in an in vitro sepsis model. Gene chip analysis and Gene Set Enrichment Analysis (GSEA) indicated that macrophage migration inhibitory factor (MIF) could be a downstream target of CSN6 in this model (Fig. 5A, B). CSN6 upregulation led to increased protein expression of both CSN6 and MIF, whereas si-CSN6 transfection significantly reduced the expression of both proteins in macrophages (Fig. 5C, D, n = 6 per group).

### MIF inhibitor reduced the effects of CSN6 on inflammation and ferroptosis of macrophage in vitro model of sepsis

**Fig. 6:**
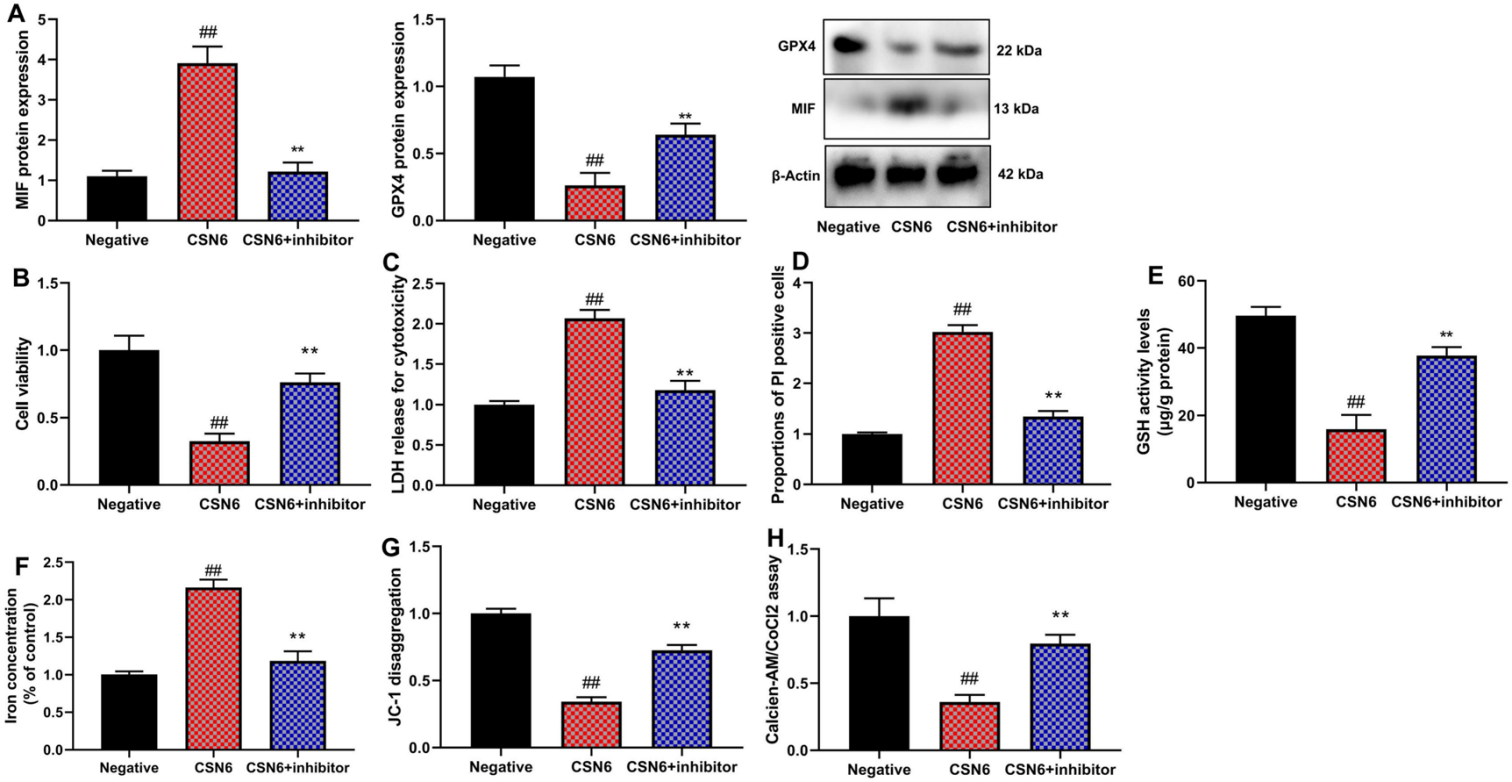
MIF inhibitor reduced the effects of CSN6 on ferroptosis of macrophage in vitro model of sepsis. MIF/GPX4 protein expression (**A**), cell proliferation (**B**), LDH activity level (**C**), PI-positive cells (**D**), GSH activity level (**E**), iron concentration (**F**), and JC-1 and calcien-AM levels (**G**, **H**). ##p < 0.01 compared to the negative group; **p < 0.01, compared to the CSN6 group.

**Fig. 7:**
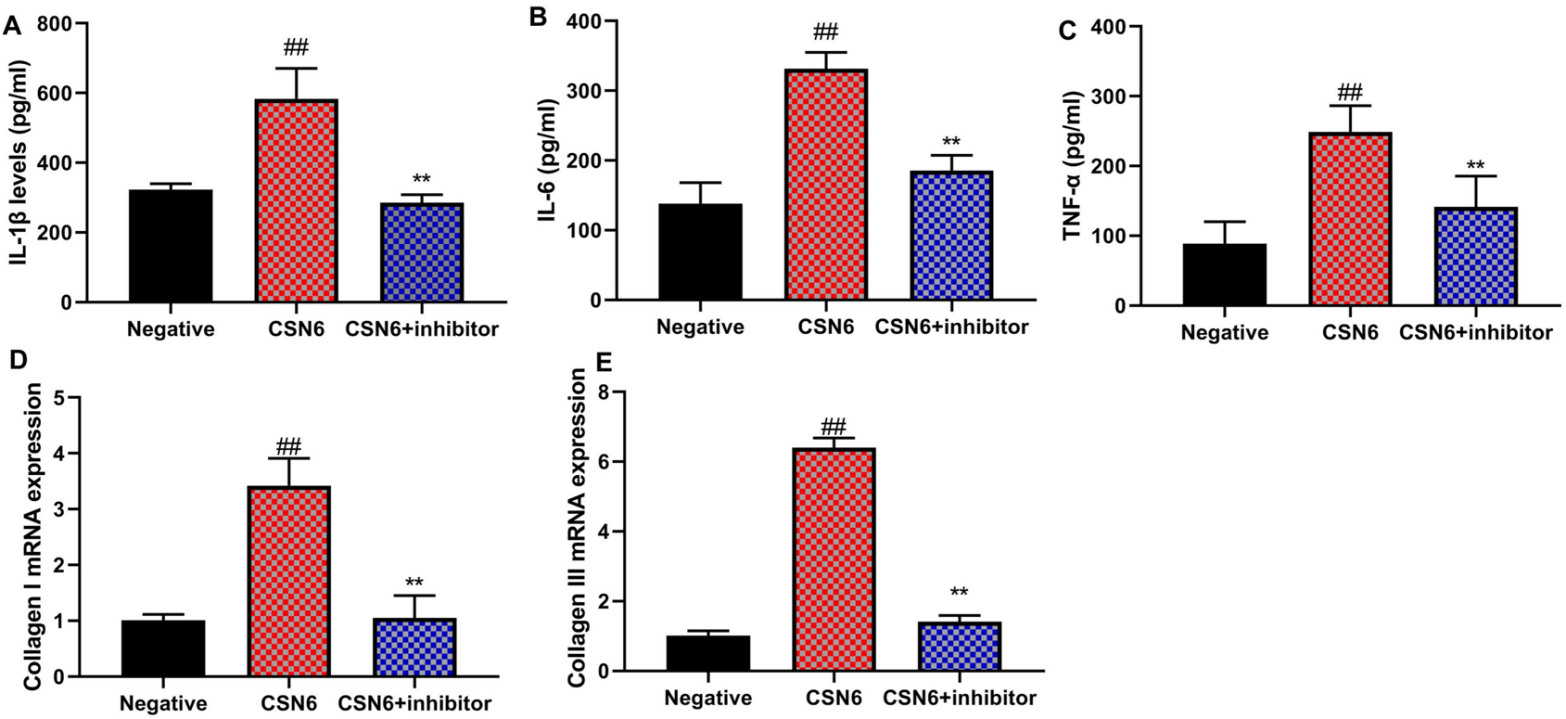
MIF inhibitor reduced the effects of CSN6 on inflammation of macrophage in vitro model of sepsis. IL-1β (**A**), IL-6 (**B**), TNF-α (**C**), and collagen I/III mRNA expressions (**D** and **E**). ##p < 0.01 compared to the negative group; **p < 0.01, compared to the CSN6 group.

The use of MIF098 (10 μM, an MIF inhibitor) attenuated the effects of CSN6 on MIF and GPX4 protein expression, cell proliferation, LDH activity, PI-positive cells, and ferroptosis (Fig. 6, n = 6 per group for protein analysis and n = 5 per group for other assays). Furthermore, the MIF inhibitor also mitigated the effects of CSN6 on the mRNA expression levels of pro-inflammatory cytokines IL-1β, IL-6, TNF-α, and collagen I/III in macrophages in an in vitro sepsis model (Fig. 7, n = 6 per group).

### CSN6 protein linked MIF protein

**Fig. 8:**
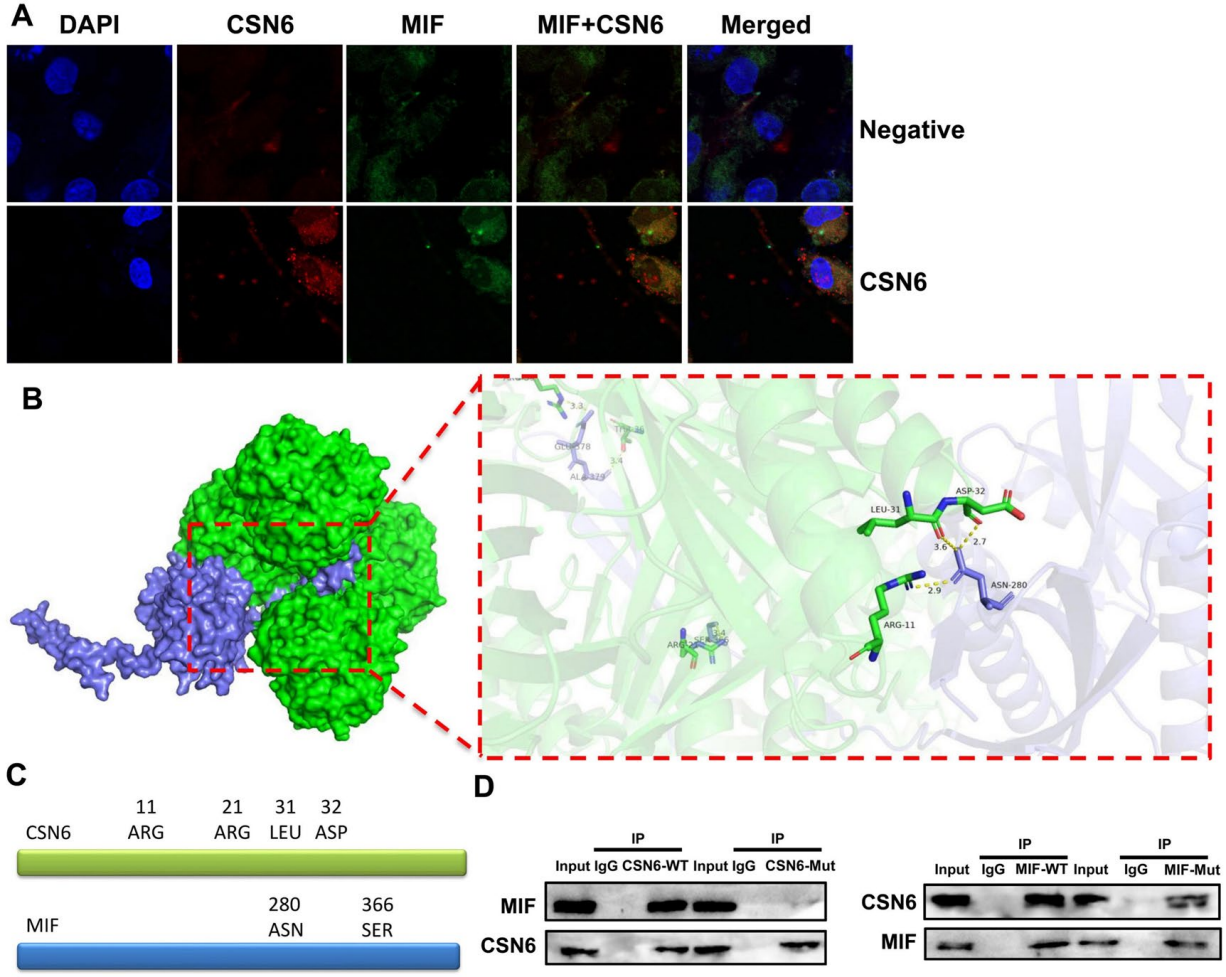
CSN6 protein linked MIF protein. Confocal microscopy (**A**), molecular docking 3D visualizd model (**B**), and Immunoprecipitation (IP) experiment (**C**). The molecular interaction between CSN6 and MIF in Fig. 8B was generated using AutoDock Vina 1.2.3 (https://vina.scripps.edu/) and visualizd with PyMOL 2.5.4 (https://pymol.org/).

This study further explored the regulatory effects of CSN6 on MIF using an in vitro macrophage model of sepsis. Confocal microscopy analysis suggested that CSN6 induces MIF expression in macrophages in an in vitro sepsis model; additional images with a higher number of cells (n = 15 fields with approximately 5–10 cells per field) were analyzed to robustly support this conclusion (Fig. 8A). The 3D model displayed CSN6 protein-linked MIF protein (Fig. 8B). The binding site of the CSN6 protein was linked to the MIF protein (Fig. 8C). IP experiments showed that CSN6 protein at 11-ARG, 21-ARG, 31-LEU, and 32-ASP linked MIF protein at 280-ASN and 366-SER (Fig. 8D, n = 3 independent experiments).

## Discussion

Sepsis is a common critical illness with a high mortality rate that significantly affects patients’ quality of life. Research has shown that the initiation factor for multiple organ dysfunction syndrome in patients with sepsis is impairment and dysfunction of the intestinal mucosal barrier, leading to dysbiosis and bacterial translocation^16^. This disruption increases intestinal permeability, allowing large amounts of inflammatory factors and endotoxins to enter the bloodstream, exacerbating the condition, and increasing mortality. Previous studies have indicated that the incidence of intestinal dysfunction in sepsis patients is approximately 50–60%, with these patients showing higher mortality rates than those with sepsis alone^17,18^. In this study, we observed increased CSN6 protein expression in macrophages in an in vitro sepsis model. There was a significant overlap between CD68 and CSN6, suggesting that CSN6 expression occurred in macrophages in the sepsis model. Wen et al. suggested that TNF-α upregulates CSN6 expression during lung inflammation^19^. These data indicate that CSN6 is involved in the progression of sepsis and inflammation.

Sepsis is a systemic inflammatory response caused by dysregulated reactions to various infections, leading to life-threatening organ dysfunction^20^. It is characterized by a high incidence and mortality rates, complex pathogenesis, and severe conditions that can result in shock. Inflammation is a common manifestation of sepsis and clinically manifests as elevated inflammatory cytokines and impaired immune functions^21^. However, the exact mechanism underlying inflammatory dysfunction in sepsis remains unclear. It is currently believed to be associated with oxidative stress, inflammatory responses, apoptosis, endotoxins, and energy metabolism^22^. Studies have shown that sustained activation of inflammation and inflammatory signaling is an early event in sepsis^23^. Our results showed that CSN6 aggravated inflammation in macrophages in an in vitro sepsis model. Mei et al. reported that CSN6 exacerbates inflammatory responses^24^. Thus, CSN6 might enhance macrophage-driven inflammation in sepsis models.

Sepsis is a systemic response to infection, leading to tissue damage and organ dysfunction, which can be life-threatening^25^. Inflammatory injury is a common manifestation of organ dysfunction in sepsis, but its pathogenesis is complex and involves oxidative stress, inflammatory responses, immune dysfunction, and mitochondrial dysfunction^26,27^. Our findings demonstrated that CSN6 aggravates ferroptosis in macrophages in an in vitro sepsis model by inducing mitochondrial damage. Wen et al. also suggested that TNF-α upregulates CSN6 expression in monocyte-derived macrophages during lung inflammation^19^. Therefore, CSN6 may exacerbate macrophage ferroptosis in sepsis models.

Macrophage migration inhibitory factor (MIF) plays a crucial role in the occurrence and development of systemic inflammatory response syndrome (SIRS). MIF is composed of 115 amino acids, is widely distributed in various organs and tissues, and is involved in the pathological processes of numerous diseases^28^. MIF is a multifunctional protein with characteristics of cytokines, neuroendocrine hormones, and enzymes^29^. It inhibits macrophage migration, promotes macrophage activation at sites of inflammation, and stimulates the secretion of cytokines, such as IL-1, IL-8, and TNF-α^30^. Its role in the inflammatory response is highly significant, making it important to measure MIF concentrations in sepsis patients for diagnostic and therapeutic purposes^31^. MIF plays a significant regulatory role in the production and release and nurse of inflammatory factors^31^. Under the influence of inflammation, plasma MIF levels exhibit varying degrees of elevation^32^. Therefore, it is crucial to measure plasma MIF concentrations during sepsis diagnosis、 treatment and nurse. In this study, we found that CSN6 induced MIF expression in macrophages in an in vitro sepsis model. Moreover, an MIF inhibitor reduced the effects of CSN6 on inflammation and ferroptosis in macrophages. We also found that CSN6 protein at residues 11-ARG, 21-ARG, 31-LEU, and 32-ASP binds to MIF protein at residues 280-ASN and 366-SER. Burger-Kentischer et al. demonstrated that CSN5 mediates MIF in mammalian cells^33^. Therefore, CSN6 may be linked to MIF to induce MIF expression in macrophages in a sepsis model.

## Supplementary Information

Supplementary Information.

## Data Availability

All data generated or analysed during this study are included in this published article [and its supplementary information files].
