# Supplementary figures and images for: CSN6 aggravates inflammation and Myocardial injury in macrophage of sepsis model by MIF
Source: Sci Rep. 2025 Jul 1;15:21527. doi: 10.1038/s41598-025-07339-1 (PMC12218151; doi:10.1038/s41598-025-07339-1)

β-actin 42 kDa

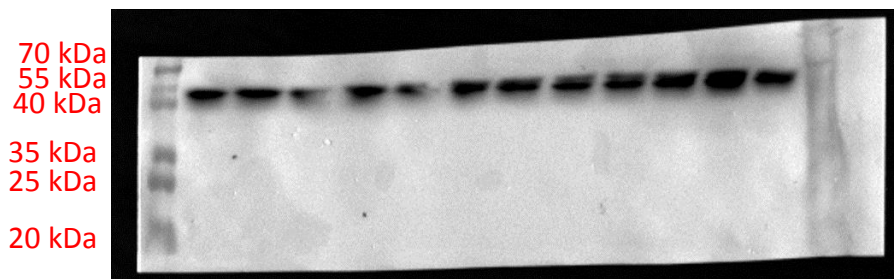

β-actin 42 kDa

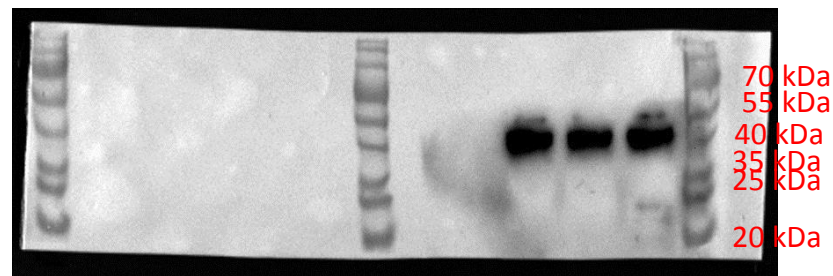

β-actin 42 kDa

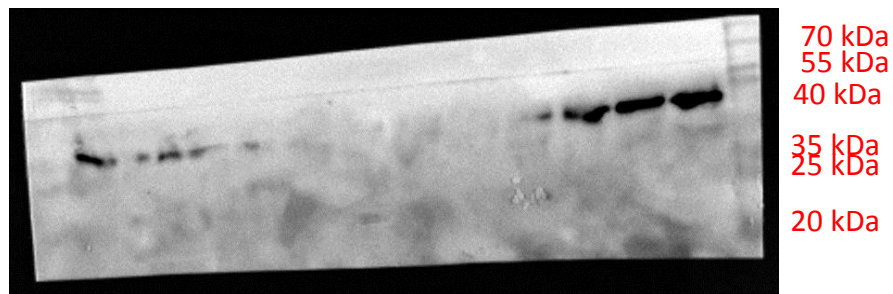

CSN 36 kDa

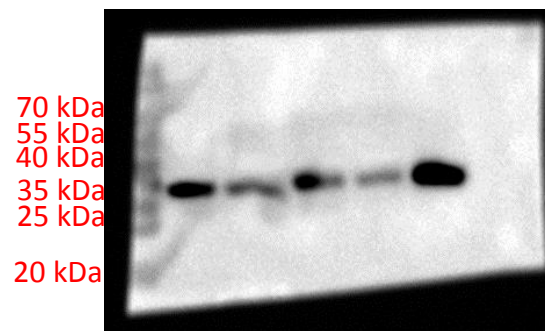

MIF 13 kDa

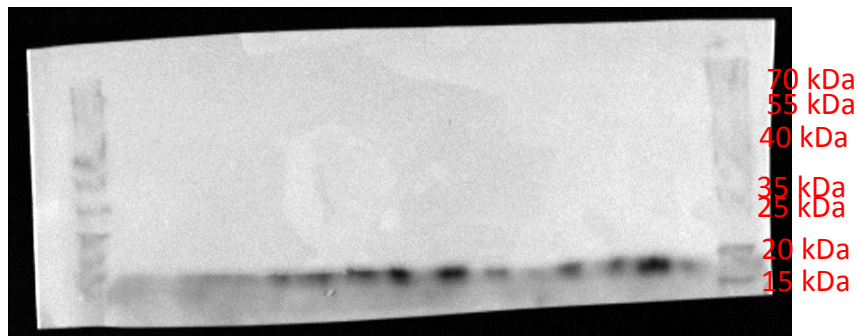

GPX4 22 kDa

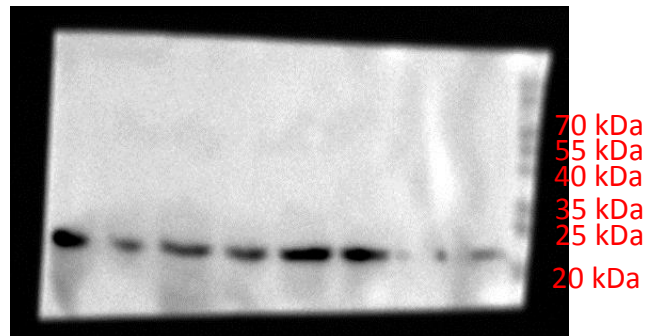

Supplement: Supplementary file 1 — Supplementary Information. [file 41598_2025_7339_MOESM1_ESM.pdf]
